# Obstacle‐Immune Microwave Wireless Power Transfer System Based on Amplitude‐Dependent Caustic Metasurface

**DOI:** 10.1002/advs.202510070

**Published:** 2025-08-04

**Authors:** Song Zhang, Hao Xue, Zhe Zheng, Xiangjin Ma, Xin Wang, Jiaqi Han, Haixia Liu, Long Li

**Affiliations:** ^1^ Key Laboratory of High‐Speed Circuit Design and EMC of Ministry of Education School of Electronic Engineering Xidian University Xi'an 710071 China; ^2^ School of Electronic Engineering Xi'an University of Posts and Telecommunications Xi'an 710100 China

**Keywords:** ADCMs, microwave wireless power transfer (MWPT), OAM beam, obstacle avoidance, RFCAB

## Abstract

Rapid advances in the Internet of Everything (IoE) drive microwave wireless power transfer (MWPT) technology for charging wireless smart devices. However, environmental obstacles and safety concerns associated with high‐power transmission present new challenges for the technology. To address these problems, an amplitude‐dependent caustic metasurface (ADCMs) design method is proposed for generating self‐bending radio frequency circular Airy beams (RFCAB). The self‐accelerating circular Airy beam has a natural hollow region, providing an obstacle avoidance and safety zone during microwave transmission focusing. The ADCMs, derived from caustic theory, are capable of generating RFCAB with arbitrary curvatures and offer flexible control over the positioning of the energy focus. An innovative feed architecture, a uniform circular array (UCA) emitting vortex electromagnetic waves, is used to match the amplitude excitation on which the metasurface depends. The designed ADCMs consists of 1/3 wavelength units and contains only two layers of substrate, with high transmittance and full 360^0^ phase coverage, ensuring efficient beam generation and manipulation. Simulation and experimental analyses demonstrate the obstacle‐avoidance characteristics and safety features under high‐power transmission of ADCMs‐based MWPT systems. The proposed MWPT system is anticipated to offer an effective obstacle avoidance and safety solution for the wireless charging of devices within the IoE.

## Graphical abstract

This paper innovatively proposes an advanced microwave wireless power transfer (MWPT) system based on the amplitude‐dependent caustic metasurface generating the circular Airy beam. The proposed MWPT system will advance the research and development of circular Airy beams in the radio frequency domain. It also provides a feasible solution to challenges such as obstacles in the environment faced by MWPT.

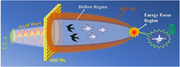

## 1 Introduction

Driven by the growth of the low‐altitude economy and the Internet of Everything (IoE), the proliferation of wireless smart devices has surged, posing a significant challenge in ensuring a continuous energy supply for these devices. Microwave wireless power transfer (MWPT) technology offers advantages such as long‐distance transmission, high power delivery, and minimal susceptibility to environmental factors, enabling flexible energy transmission over a wide range.^[^ ^1^ ^]^ The study of the application of MWPT technology in the IoE and low‐altitude economy can provide a reliable and effective solution to the above challenges. Currently, MWPT is primarily implemented using antenna arrays and large‐scale parabolic antennas,^[^ ^2^, ^3^ ^]^ which introduce additional challenges in feed network and circuit design, increasing system complexity and cost, and making integration with other devices more difficult. Meanwhile, in the existing MWPT system, the energy transmission beams are mainly high‐gain beams,^[^ ^4^, ^5^ ^]^ Bessel beams,^[^ ^6^, ^7^ ^]^ and focused beams based on Gaussian amplitude excitation.^[^ ^8^, ^9^ ^]^ In the wireless charging technology utilizing the aforementioned beams, the energy is continuously distributed and propagated between the transmitter and receiver. Therefore, in practical applications, energy transmission cannot bypass obstacles between the transmitter and receiver, nor can it guarantee a safe area during high‐power transmission. Airy beams with self‐bending propagation have been studied as a solution to the issue of obstacles in microwave energy transmission.^[^ ^10^, ^11^, ^12^, ^13^, ^14^ ^]^ However, the energy of an Airy beam is concentrated along its curved propagation path, and it gradually diminishes as the beam propagates. As a result, only a small fraction of the energy is captured by the receiver, which is detrimental to the efficiency of wireless charging. Circular Airy beam in the radio frequency (RF) field offers a potential approach to solving microwave wireless charging in complex environments. When transmitting microwave energy, it can form a hollow region between the transmitter and receiver. Obstacles in this region have minimal impact on the efficiency of energy transmission, and the region can also provide a safe area for high‐power microwave transmission.

At present, the design and implementation of circular Airy beams are primarily based on the principles of Fourier optics,^[^ ^15^, ^16^, ^17^, ^18^, ^19^, ^20^, ^21^ ^]^ requiring equipment such as Gaussian lasers, spatial light modulators (SLM), and Fourier lenses, along with precise spatial arrangement for experimental generation. This results in complex, bulky, and large‐generation systems, which limit the practical application of circular Airy beams. In the RF field, the design for generating a circular Airy beam is mainly based on the initial beam field in the existing works.^[^ ^22^, ^23^ ^]^ However, this method only involves binary phases (0 and π) modulation. Due to the limited precision of the modulation phase, the focused energy of the circular Airy beam is weakened. The caustic theory based on geometric optics has been widely applied to generate Airy beams,^[^ ^24^, ^25^, ^26^, ^27^ ^]^ enabling an increase in beam intensity while facilitating the generation of self‐bending beams with arbitrary curvature. When applied to the design of radio frequency circular Airy beam (RFCAB), this theory simplifies the beam generation system. Through continuous phase modulation, the focused intensity can be increased, while also enabling flexible control of the focal position according to the location of different receiver devices. The electromagnetic metasurface has emerged as a highly effective platform for the precise manipulation of both the amplitude and phase profiles of electromagnetic waves.^[^ ^28^, ^29^, ^30^, ^31^, ^32^ ^]^ This capability enables unparalleled flexibility in beam generation and control, while preserving the benefits of low complexity and a compact profile. This technology has found extensive application in the generation of diverse electromagnetic beams, such as Airy beams,^[^ ^33^ ^]^ Bessel beams,^[^ ^34^ ^]^ and in various MWPT systems.^[^ ^35^, ^36^, ^37^, ^38^, ^39^, ^40^ ^]^ By integrating caustic theory with metasurface, the resulting caustic metasurface leverages the advantages of both to offer unprecedented convenience in generating RFCAB. This approach also provides an efficient and reliable solution for microwave power transfer applications utilizing RFCAB. However, the caustic metasurface requires a specific amplitude dependence, which cannot be satisfied by the Gaussian beam feed typically used in conventional metasurfaces. The orbital angular momentum (OAM) beams exhibit a distinctive radiation amplitude distribution, which precisely meets the amplitude excitation required by caustic metasurfaces, thereby enabling the efficient conversion of the OAM beam into a circular Airy beam for wireless energy transmission.

**Figure 1:**
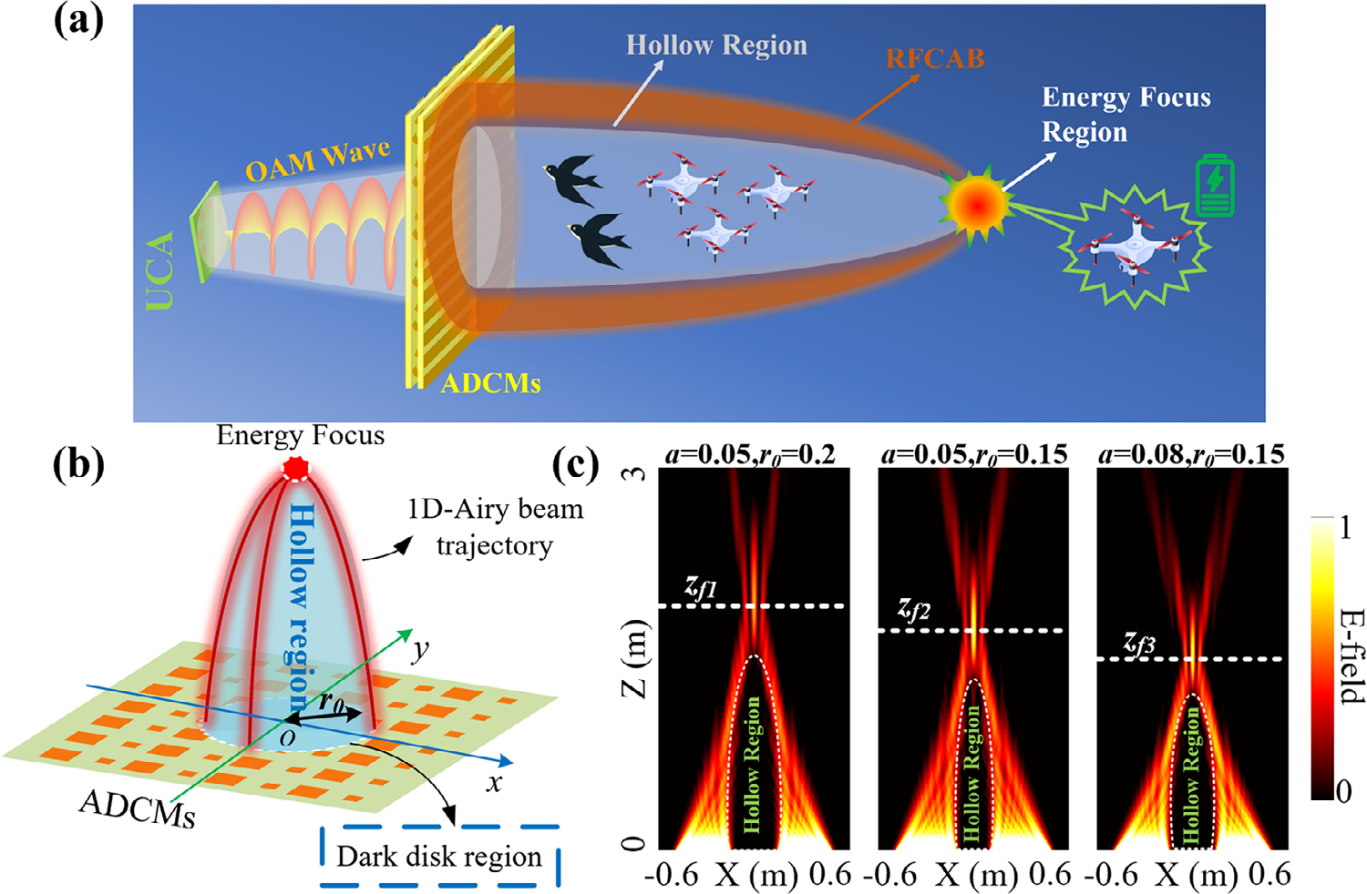
a) The scenario diagram of the proposed obstacle‐avoiding MWPT system based on the ADCMs. b) The illustration diagram for generating an RFCAB based on the ADCMs. c) The normalized E‐field distribution on the vertical section of the RFCAB corresponds to different values of *a* and *r_0,_* where the array size is 1.2 m and the results are obtained by MATLAB.

In this paper, we aim to design an advanced MWPT system based on an amplitude‐dependent caustic metasurface (ADCMs), so as to provide an obstacle‐avoiding and safe wireless charging scheme for microwave wireless power transmission in complex environments. The design of the ADCMs aims to generate an RFCAB that bends and propagates, focusing energy at the endpoint. This establishes an obstacle‐avoidance and safety zone between the transmitter and receiver while minimizing electromagnetic interference to nearby smart devices or unmanned aerial vehicles (UAV). The integration of caustics theory with the metasurface in ADCMs significantly reduces the complexity of the beam generation system while substantially enhancing the flexibility of beam design and manipulation. Furthermore, due to the amplitude‐dependent characteristics of the ADCMs, a uniform circular array (UCA) emitting OAM beams is chosen as the optimal feed for the ADCMs. This configuration ensures the appropriate amplitude excitation, allowing most of the energy emitted by the feed to be effectively utilized in generating the RFCAB. Moreover, the designed ADCMs unit has a sub‐wavelength size (1/3 of the wavelength) and retains a high transmission efficiency (>80%) while offering 360° phase coverage. This enables robust strength for the continuous phase manipulation capability of the ADCMs. The scenario of the obstacle‐avoiding MWPT system designed by ADCMs is shown in **Figure** **1**a. To validate the proposed method, an ADCMs operating at 10 GHz, comprising 51 × 51 units, was designed and fabricated. The simulation and experimental results demonstrate that the proposed MWPT system is capable of circumventing obstacles and establishing a safe zone. The designed ADCMs‐based MWPT system offers advantages such as a low profile and ease of integration. It is expected to provide a feasible solution for obstacle‐avoiding and safe microwave wireless energy transfer, particularly for wireless devices in the IoE and UAV swarms within the low‐altitude economy.

## 2 Results

### 2.1 Design and Generation of the RFCAB

The RFCAB can be viewed as a radial superposition of multiple self‐bending 1D Airy beams generated by the ADCMs, resulting in a caustic surface that converges to an energy focus at the endpoint. Additionally, to ensure that the RFCAB, designed based on the caustic theory, includes a hollow region (for safety and obstacle avoidance), the array must feature a dark disk region that generates extremely low beam energy. Figure 1b illustrates the schematic diagram for generating an RFCAB by using an ADCMs, where the radius of the dark disk region is denoted as *r_0_*. Therefore, the caustic phase distribution required for the metasurface to generate an RFCAB can be obtained as shown in Equation (1), and the derivation details are given in Note S1 (Supporting Information).

(1) $$\phi_{cu}=\frac{4k_{0}\sqrt{a}}{3}{\left(\sqrt{x_{m}^{2}+y_{m}^{2}}-r_{0}\right)}^{\frac{3}{2}}$$

where (*x_m_*,*y_m_*) represent the coordinates of the ADCMs unit and *a* is the curvature of the beam. Different values of *r_0_* and *a* will affect the focal length (*z_f_*) and the hollow region size (*V_Hollow_*) of the RFCAB as shown in Equations (S6) and (S7) (Supporting Information). Figure 1c shows the normalized electric field (E‐field) distribution on the vertical plane of RFCAB under different combinations of *a* and *r_0_*. As shown in the figure, the RFCAB features a very low field distribution in a hollow region during propagation. The obstacles in this region will have minimal impact on energy transmission, and the region also provides a safe zone for high‐power microwave energy transmission. Furthermore, by adjusting the design parameters *a* and *r_0_*, the focal position and hollow characteristics of the RFCAB can be flexibly manipulated. Here, to satisfy the amplitude excitation required by the ADCMs, a uniform circular array (UCA) integrated with OAM beams is proposed as the optimal driving source for the array. This is due to the inherent divergence characteristics of the OAM wavefronts, which precisely match the dark disk region of the array. The geometric configuration of the UCA, particularly its radius *R*, and the mode *l* characterizing the OAM beam, jointly determine the amplitude excitation profile on which the ADCMs depend, as shown in Figure S4 (Supporting Information). The design principles of the UCA, along with the effects of *R* and *l* on the radiation intensity of the OAM beams, are provided in Note S2 (Supporting Information).

**Figure 2:**
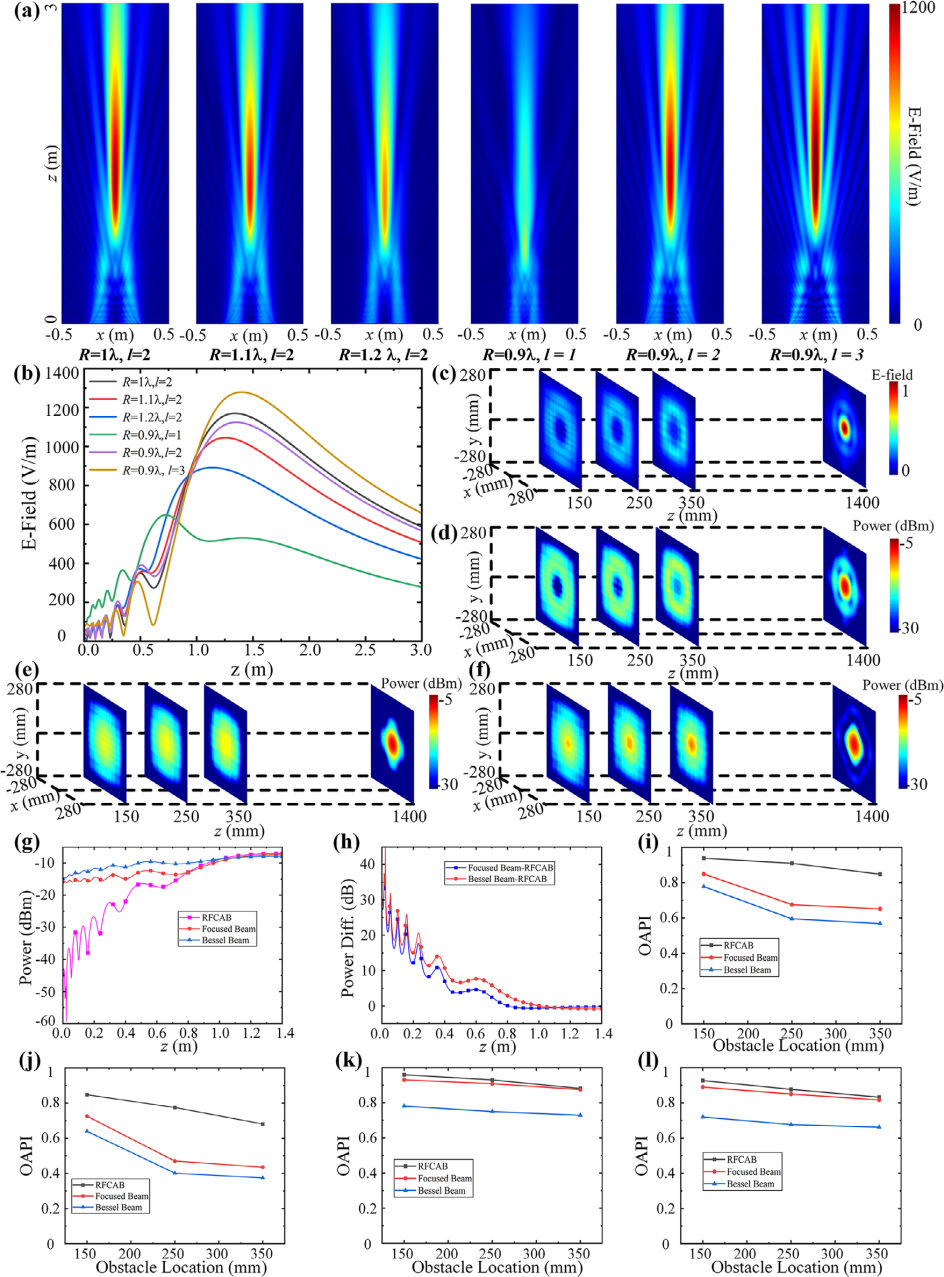
a). The field distribution of the RFCAB under UCA with different radius *R* and OAM beams with different modes *l*. b) The E‐field value along the vertical central axis of the RFCAB. c) The normalized E‐field distributions, d) The power distributions of RFCAB on the different horizontal planes. The power distributions of e) the focused beam, f) the Bessel beam on the different horizontal planes. g) The power distribution along the central axis of RFCAB, focused beam, and Bessel beam. h) The power difference along the central axes between RFCAB and the focused beam and the Bessel beam. The OAPI of the three beams after adding metal plate obstacle with the size i) 100 mm × 100 mm, and j) 140 mm × 140 mm at various positions. The OAPI of the three beams after adding wooden board obstacle with the size k) 100 mm × 100 mm, and l) 140 mm × 140 mm at various positions.

**Figure** **2**a illustrates the field distribution of the RFCAB on the vertical observation plane, generated based on the ADCMs, where UCA with different radius *R* and OAM beams with different modes *l* are used as the driving sources of the ADCMs. The generation details for these results are provided in Note S3 (Supporting Information). Figure 2b shows the variation in the E‐field value along the vertical central axis of the RFCAB as a function of the z‐coordinate. As observed in Figure 2a,b, different values of *R* and *l* affect the focus characteristics (position and intensity) and the hollow region characteristics of the RFCAB. This is due to the fact that changes in *R* and *l* alter the distribution of excitation amplitudes on which the ADCMs depends, as illustrated in Figures S5 and S6 (Supporting Information).

Specifically, as *R* increases and *l* decreases, the excitation amplitude distribution contracts inward. Conversely, when *R* decreases and *l* increases, the excitation amplitude distribution expands outward. This behavior is consistent with the description provided in Equation S9 (Supporting Information). Due to the varying amplitude excitations caused by changes in *R* and *l*, the phase contributions from different regions of the ADCMs to the RFCAB are altered, thereby affecting the focusing and hollow region characteristics. This will also introduce additional degrees of freedom for regulating the RFCAB characteristics generated by the ADCMs.

### 2.2 Transmission Characteristics of RFCAB in MWPT

For the application of MWPT, the transmission characteristics of RFCAB in the case of *R =* 0.9 λ and *l* = 2 in Figure 2a are analyzed. Figure 2c illustrates the normalized electric field distribution on the horizontal planes at distances of 150 mm, 250 mm, and 350 mm from ADCMS, as well as on the focusing plane of the RFCAB (z = 1400 mm). As shown in the figure, before energy focusing, distinct hollow regions are observed on the different horizontal planes. Moreover, the electric field in the hollow region on the same observation plane is significantly lower than that in the surrounding other areas. Figure 2d presents the corresponding power distribution on different horizontal planes. From the figure, the power distribution in the hollow regions at z = 150 mm and 250 mm can be as low as more than 14 dB compared to the power in other surrounding areas on the same horizontal observation plane. Furthermore, as the RFCAB propagates, with the reduction of the hollow region and the enhancement of beam interference, the power in the hollow region at z = 350 mm can also reach as low as about8 dB compared to the surrounding area. From a longitudinal energy distribution perspective, the power in the hollow regions located at z = 150, 250, and 350 mm can be as low as 23.9, 24.7, and 17 dB, respectively, compared to the power at the energy focus (z = 1400 mm). These results highlight the hollow characteristics of the RFCAB and demonstrate its potential to create a safe zone during high‐power microwave transmission.

To further validate the characteristics and advantages of RFCAB, it is compared with two existing near‐field energy transmission beams: the focused beam and the Bessel beam. The details for generating the focused beam and Bessel beam are provided in Note S4 (Supporting Information). To ensure a fair comparison, the array apertures used to generate the RFCAB, focused beam, and Bessel beam are identical, and the locations of the strongest energy point are also set the same. Figure S7b–d (Supporting Information) presents the normalized electric field distributions on the vertical planes of the three beams. To verify the hollow characteristics of RFCAB, the electric field distributions along the central axes of the beams (indicated by the white lines in Figure S7, Supporting Information) were compared, as shown in Figure S8 (Supporting Information). As observed, before reaching the maximum field value along the white line, the electric field in the hollow region of RFCAB is significantly lower than that of the focused beam and Bessel beam at the corresponding position. Furthermore, the power distributions on different horizontal planes for the generated focused beam and Bessel beam were analyzed, as shown in Figure 2e,f. A comparison with Figure 2d reveals that RFCAB exhibits distinct hollow characteristics compared to both the focused beam and the Bessel beam. Figure 2g shows the power distribution along the central axis before the focus for the three beams. Figure 2h illustrates the power difference along the central axes between RFCAB and the focused beam and the Bessel beam. As observed, the power in the hollow region of RFCAB can be more than 10 dB lower than that of the focused beam and Bessel beam. This clearly demonstrates the advantage that RFCAB can provide a safe area when transmitting high‐power microwave energy compared to the focused beam and Bessel beam.

Due to the extremely low field intensity in the hollow region of RFCAB, obstacles within this area can be bypassed during microwave energy transmission. To quickly evaluate the obstacle avoidance capability of RFCAB and compare it with that of the focused beam and Bessel beam, the current source simulation was implemented in Altair FEKO. The details of the simulation are provided in Note S5 (Supporting Information). Tables S1 and S2 (Supporting Information) present the power at the focal planes of the three beams after introducing metal plate obstacles of dimensions 100 mm × 100 mm and 140 mm × 140 mm at z = 150, 250, and 350 mm, respectively. For ease of analysis, the obstacle avoidance performance index (OAPI) is proposed to quantify the obstacle avoidance capability of the beams, and its calculation method is shown in Equation (2).

(2) $$OAPI=\frac{P_{obs}}{P_{0}}$$

where *P_obs_* represents the power at the focus with an obstacle present, and *P_0_* represents the power at the focus without adding an obstacle. The closer the OAPI is to 1, the better the obstacle avoidance capability of the beam is. The OAPI of the three beams after adding 100 mm × 100 mm and 140 mm × 140 mm metal obstacles is shown in Figure 2i,j. As observed from the figure, after adding metal obstacles of different sizes at various positions, the OAPI of RFCAB is higher than that of both the focused beam and the Bessel beam. This difference becomes more pronounced as the position of the metal obstacles increases. Subsequently, wooden board obstacles of different sizes (100 mm × 100 mm and 140 mm × 140 mm) were added to various positions. The power variations at the focus after introducing the wooden board obstacles are presented in Tables S3 and S4 (Supporting Information), while the OAPI of the three beams is shown in Figure 2k,l. As seen from the figures, after changing the obstacle from metal to wooden, the OAPI of all three beams has improved. However, the OAPI of the RFCAB beam remains higher than that of both the focused and Bessel beams. From Figure 2i–l, it can be concluded that, compared to existing energy transmission beams, the RFCAB‐based MWPT system demonstrates superior obstacle avoidance capabilities, especially in the presence of metal obstacles, where this ability is more pronounced. Furthermore, regardless of whether the obstacle is metal or wooden, energy transmission based on RFCAB exhibits excellent obstacle avoidance performance when compared to the Bessel beam.

To better demonstrate the obstacle avoidance performance of RFCAB, the improvement index (EI) is defined to quantify the improvement rate of the obstacle avoidance capability of the MWPT system based on RFCAB when facing obstacles in the environment compared with the MWPT systems of focused beams and Bessel beams. Tables S5–S8 (Supporting Information) present the EI of the RFCAB‐based MWPT system, compared to the focused beam and Bessel beam, under different obstacle positions and materials. As shown in the tables, in the presence of metal obstacles, the obstacle avoidance ability of the RFCAB‐based MWPT system can be increased by 64.72% compared with the focused beam, and by 93.17% compared with the Bessel beam. In contrast, when non‐metallic obstacles are present, the improvement in obstacle avoidance capability of the RFCAB‐based MWPT system is modest compared to the focused beam, at 3.7%, whereas compared to the Bessel beam, its obstacle avoidance capability can be improved by 20.71%.

To summarize, the above results demonstrate that the RFCAB, due to its hollow region characteristics, provides a reliable solution to the problem of obstacles in the microwave wireless energy transmission environments. Additionally, it offers a safe zone for high‐power microwave transmission. By comparing with the focused beam and the Bessel beam, the microwave energy transmission characteristics of RFCAB have been further validated. While the obstacle avoidance capability of RFCAB does not show significant improvement in the presence of non‐metallic obstacles compared to the focused beam, its hollow region characteristics still provide a considerable advantage in ensuring safe high‐power microwave energy transmission.

### 2.3 Model Design, Simulation and Measurement

Here, a prototype of an obstacle‐immune MWPT system based on RFCAB will be designed and constructed using a UCA integrated with an OAM beam that excites the ADCMs. Without loss of generality, the RFCAB in the case of *R* = 0.9 λ and *l* = 2 shown in Figure 2a will be generated by the designed system prototype.

#### 2.3.1 UCA

**Figure 3:**
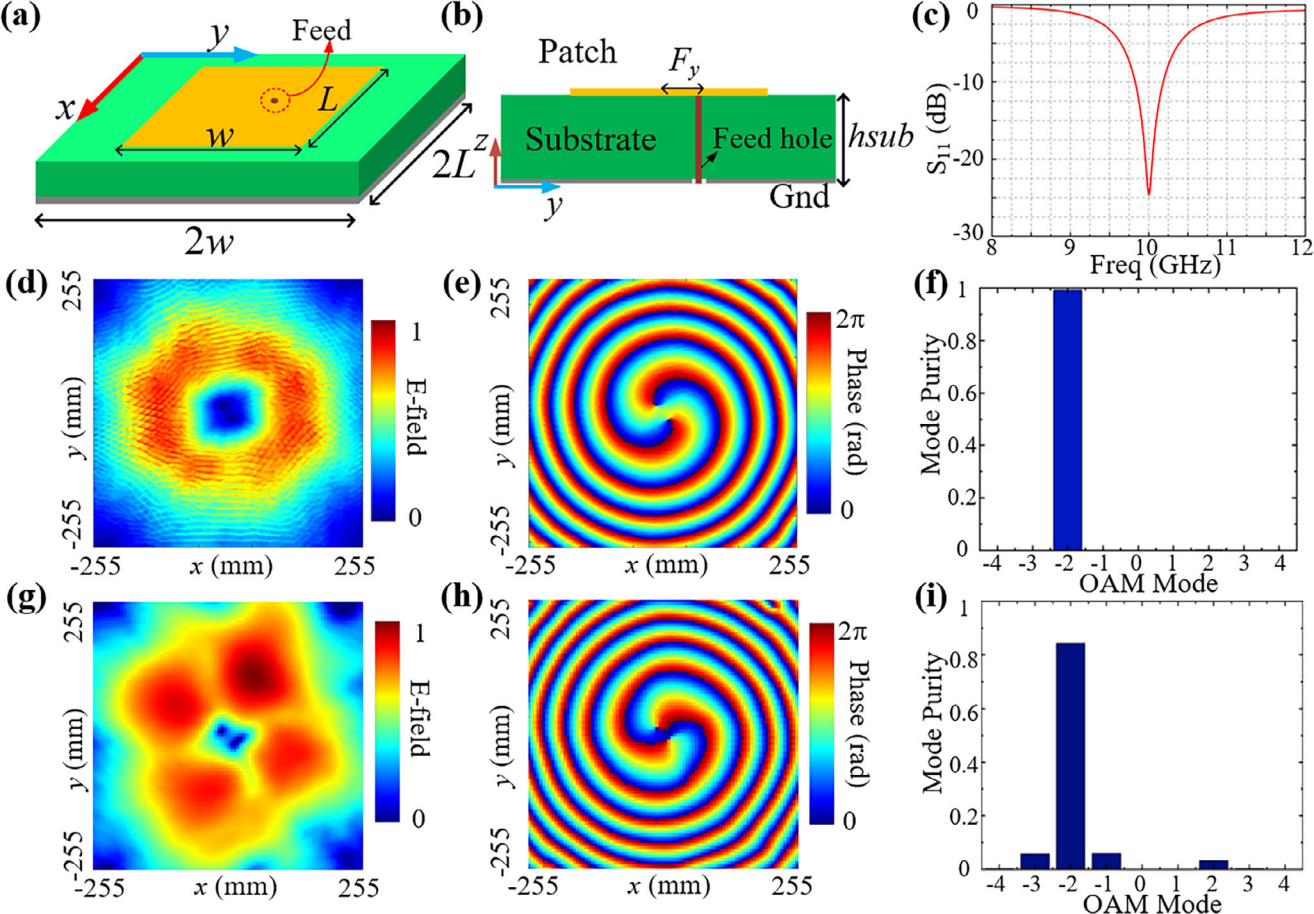
a,b) The geometric structure of the UCA unit. c) The reflection coefficient (S_11_) of the unit. d,e) The simulated amplitude and phase distributions of the UCA on the horizontal observation plane located 240 mm away from the UCA. f) The mode purity of the OAM beam obtained through simulation. g,h) The measured amplitude and phase distributions of the UCA on the horizontal observation plane located 240 mm away from the UCA. i) The measured mode purity of the OAM beam.

The UCA unit, shown in **Figure** **3**a,b, consists of a rectangular radiation patch at the top and a substrate with the ground, where the dielectric constant of the substrate is 2.5. The detailed design parameters and description of the UCA unit are given in Note S6 (Supporting Information). It operates at a frequency of 10 GHz and works in the *y*‐polarization state. Figure 3c presents the reflection coefficient (S_11_) of the unit, which is obtained through simulations using ANSYS HFSS. As illustrated, the unit has an extremely low S_11_ at 10 GHz, indicating that it is well‐matched to the feed.

In order to simplify the design of the UCA array that generates *l* = ‐2 mode OAM waves, 8 units are used here to discretize the required phase, with the phase of each unit calculated according to Equation S8 (Supporting Information). The units are arranged on a circle with a radius of *R* = 0.9 λ, as shown in Figure S11a (Supporting Information). A feeding network, depicted in Figure S11b (Supporting Information), is used to supply power to the UCA units. Between the feeding network and the UCA array's ground layer, there is a dielectric layer with a height of 0.93 mm and a dielectric constant of 2.5. The constructed UCA array, presented in Figure S11 (Supporting Information), demonstrates the advantages of compact geometry, low‐cost fabrication, and low‐profile configuration.

To meet the amplitude excitation dependent upon by ADCMs, the distance between the UCA and the metasurface is set to 240 mm. The E‐field distribution on the horizontal observation plane, located 240 mm from the UCA, is obtained through simulations in ANSYS HFSS, as shown in Figure 3d,e. The size of the observation plane is 510 mm × 510 mm, which matches the size of the ADCMs. It can be seen from Figure 3d,e that the amplitude distribution has an extremely low field region at the center, and the phase has a helical phase distribution, which are the unique characteristics of the OAM beam. Figure 3f shows the mode purity distribution for the generated OAM beam, illustrating that the OAM beam corresponds to the *l* = −2 mode and exhibits high mode purity, as intended in the design. To validate the correctness of the design, the UCA prototype was fabricated and measured in a microwave anechoic chamber, as shown in Figure S12 (Supporting Information). Using planar near‐field scanning technology, a moving probe was employed to scan a plane 240 mm from the UCA. The amplitude and phase distributions of the electric field in this plane were then obtained, as illustrated in Figure 3g,h. Figure 3i shows the measured mode purity distribution of the generated OAM beam. As shown in the figure, the amplitude and mode purity distributions of the measured results exhibit some discrepancies compared to the simulation results. These differences are primarily attributed to machining errors in the UCA prototype, as well as measurement uncertainties associated with the microwave anechoic chamber and equipment. Nevertheless, the measurement results still confirm that the designed UCA successfully generates an OAM beam in the desired *l* = ‐2 mode and presents the characteristics of the amplitude null and spiral phase of the OAM beam.

#### 2.3.2 ADCMs Design and RFCAB Generation

**Figure 4:**
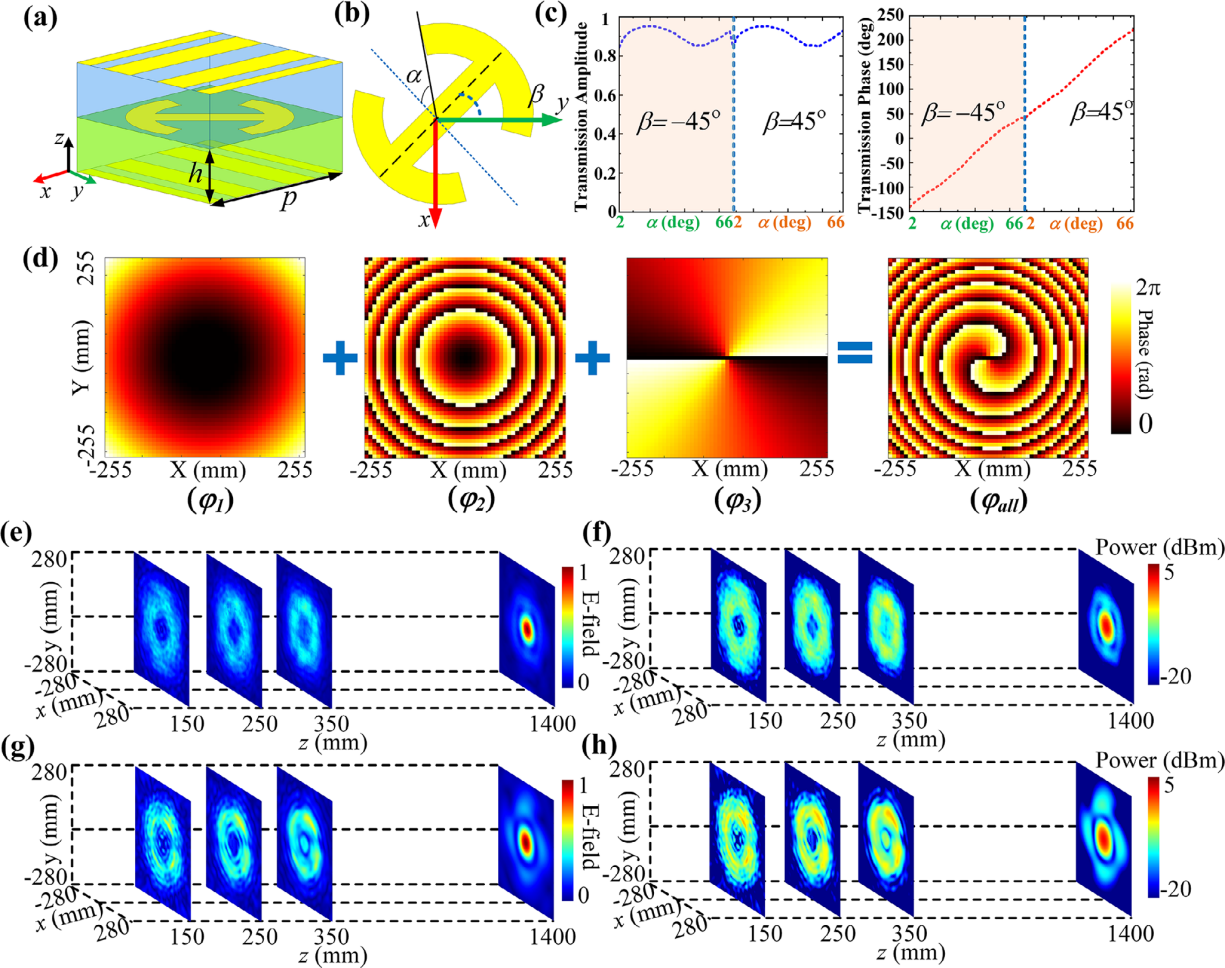
a,b) The geometric structure of the ADCMs unit. c) The amplitude and phase manipulation characteristics of the unit corresponding to parameters *α* and *β*. d) The required phase distributions for designing the ADCMs. e,f) The simulated normalized E‐field and power distributions on various planes at different distances from ADCMs. g‐h) The measured normalized E‐field and power distributions on various planes at different distances from ADCMs.

The designed ADCMs is a transmissive metasurface, and its unit is shown in **Figure** **4**a. The unit period is *p* = 10 mm, and it is composed of two layers of metal polarization grid and a metal patch with polarization conversion function in the middle. It consists of only two layers of F4B substrate with a dielectric constant of 2.65, a loss angle of 0.001, and a height of the dielectric layer is *h* = 3 mm. The improved I‐shape metal structure of the intermediate layer is shown in Figure 4b.^[^ ^41^ ^]^ *α* represents the size of the metal ring opening, which influences the distribution of the induced current on the metal, and *β* represents the rotation angle of the I‐shape structure relative to the *y*‐axis, which is used to change the direction of the induced current.

An electromagnetic polarization conversion function can be achieved by adjusting *β* = 45° or *β* = ‐45°. When the *y*‐polarized beam is incident from above onto the unit shown in Figure 4a, the induced current distribution and direction on the interlayer metal can be modulated by adjusting the parameters *α* and *β* of the I‐shaped structure, thereby enabling phase manipulation of the converted *x*‐polarized beam. Figure 4c illustrates the manipulation characteristics of the amplitude and phase of the designed ADCMs unit. As shown in the figure, a 360° beam phase control capability can be achieved while maintaining a transmission efficiency greater than 80% by adjusting various combinations of *α* and *β*. The upper and lower polarization grids ensure the regulation of electromagnetic waves with specific polarization and avoid the interference of electromagnetic beams with other polarizations. Compared with other MWPT transmission metasurface units,^[^ ^9^, ^34^, ^40^ ^]^ the designed ADCMs unit has the advantages of compactness and low profile while realizing the beam phase control capability of 360° and high transmittance, and is more conducive to the integration with other devices, building an efficient MWPT system.

For generating the desired RFCAB, the ADCMs array is designed with a size of 510 mm × 510 mm (17 λ × 17 λ), consisting of 51×51 units. The array parameter *r_0_* is 75 mm, and the beam parameter *a* is 0.02 (Details in Note S3, Supporting Information), so the caustic phase distribution of the ADCMs can be obtained according to Equation (1), as shown in Figure 4d (*φ_1_*). Since the UCA is 240 mm away from the ADCMs for providing the necessary amplitude excitation, the path delay phase caused by the feed position needs to be compensated by the units according to Equation S13 (Supporting Information) as shown in Figure 4d (*φ_2_*). For concentrating energy into the focus, the vortex phase of the OAM beam emitted by the UCA is also required to be compensated by the ADCMs, as shown in Figure 4d (*φ_3_*). Consequently, the total phase distribution necessary for designing the ADCMs is presented in Figure 4d (*φ_all_*). By the relationship between phase and unit parameters, the constructed metasurface is shown in Figure S13a (Supporting Information), and the distribution of patches in the middle layer of the array is presented in Figure S13b (Supporting Information).

Based on the designed ADCMs and UCA models, an efficient RFCAB generation system is composed, and the normalized electric field distribution of RFCAB on the vertical plane can be obtained through simulation in ANSYS HFSS, as shown in Figure S14 (Supporting Information). Additionally, the horizontal electric field distribution of the RFCAB is analyzed by constructing observation planes parallel to the metasurface at z = 150, 250, and 350 mm, and at the focal point (z = 1400 mm), as shown in Figure 4e. As depicted in the figure, the designed ADCMs successfully generated RFCAB with a distinct hollow characteristic. Figure 4f presents the simulation results of power distribution on various horizontal planes. The data reveals that the power within the hollow region at z = 150 and 250 mm can be as low as more than 10 dB compared to the power in other surrounding areas on the same horizontal observation plane. With the propagation of the beam, the power distribution in the hollow region at z = 350 mm can also reach as low as more than about7 dB compared to the surrounding area. These results differ slightly from those presented in Figure 2d, which can be attributed to enhanced mutual coupling between the ADCMs units, beam interference effects, and potential model construction errors. Despite some allowable discrepancies, the simulation results confirm the accuracy of the designed RFCAB generation system.

Furthermore, the prototype of the RFCAB generation system was fabricated and measured in a microwave anechoic chamber, as shown in Figure S15 (Supporting Information). Similar to UCA measurement, the normalized electric field distribution on the observation planes at distances of 150, 250, 350, and 1400 mm from the ADCMs was obtained using planar near‐field scanning technology, as shown in Figure 4g. Figure 4h presents the measurement results of the power distribution on different horizontal planes. By comparing Figure 4g,h with Figure 4e,f, both the simulation and measurement results demonstrate the consistency between the electric field and power distributions. These findings highlight the microwave energy transmission characteristics of the RFCAB and confirm the accuracy of the RFCAB generation system design, which includes the UCA and ADCMs. More importantly, the results unequivocally validate the feasibility and effectiveness of the proposed methodology for RFCAB generation, based on the ADCMs excited by the UCA architecture integrated with OAM beams.

#### 2.3.3 MWPT System Based on the ADCMs Generating RFCAB

**Figure 5:**
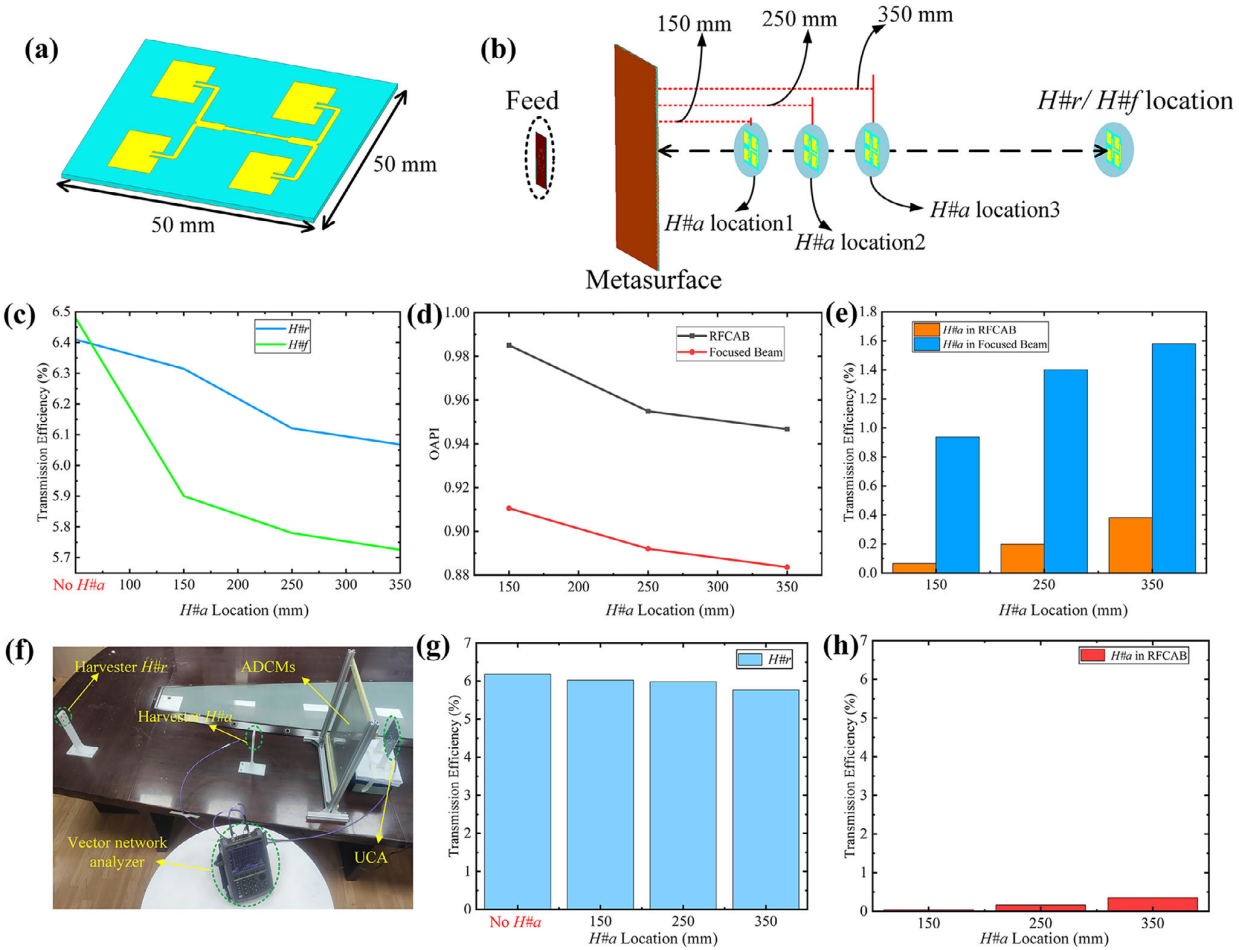
a). The designed energy harvester based on the microstrip antenna array. b) The schematic diagram of the MWPT system setup after adding another harvester. c) Effect of adding *H#a* at different positions on DC‐to‐DC transmission efficiency of *H#r* and *H#f*. d) The variation of the OAPI in the two MWPT systems with the addition of *H#a* at different positions. e) The DC‐to‐DC transmission efficiency of *H#a* at different positions in MWPT systems. f) The implemented MWPT system utilizing the RFCAB generated by ADCMs. g) The measured DC‐to‐DC transmission efficiency of *H#r* after adding *H#a* at different locations. h) The measured DC‐to‐DC transmission efficiency of *H#a* at different locations in the hollow region of the RFCAB.

Here, an advanced MWPT system based on ADCMs generating RFCAB is constructed by adding an energy harvester composed of 2 × 2 microstrip antennas at the focal point (*z_f_* = 1400 mm). **Figure** **5**a is the designed microstrip antenna array harvester, and its size is 50 mm × 50 mm. From Figure S16b (Supporting Information), the reflection coefficient of the harvester shows a good match. The schematic diagram of the constructed MWPT system is shown in Figure S16c (Supporting Information). Through simulation in ANSYS HFSS, the obtained DC‐to‐DC microwave energy transmission efficiency is 6.41% at the focus of RFCAB. Compared with the existing MWPT system based on the focused beam and Bessel beam,^[^ ^34^, ^36^ ^]^ the DC‐to‐DC efficiency based on RFCAB obtained here has certain advantages.

Furthermore, to verify the characteristics of the novel MWPT system, another microwave energy transfer system, based on the same metasurface aperture generating the focused beam of Figure 2e, was constructed for comparison. By adding the same harvester at the focal point (same as the RFCAB, *z_f_* = 1400 mm) of the focused beam, the DC‐to‐DC energy transmission efficiency is 6.48%. Additionally, in both energy transfer systems (based on RFCAB and focused beam), another identical harvester is positioned at the location corresponding to the hollow region of RFCAB. This setup is designed to assess the obstacle‐avoidance capability and the hollow characteristics of the RFCAB in comparison to the focused beam.

The additional harvester is named *H#a*, and the harvesters located at the focal point of the RFCAB and the focused beam are named *H#r* and *H#f*, respectively. The locations of the *H#a* are 150, 250, and 350 mm from the metasurface, as shown in Figure 5b. Figure 5c shows how the DC‐to‐DC transfer efficiency of *H#r* and *H#f* changes with the position of *H#a* that has been added, and Figure 5c is the OAPI of the two MWPT systems. The results show that the obstacle (represented by *H#a*) has a lesser impact on the receiving efficiency at the focal point of the RFCAB compared to the focused beam, proving that the MWPT system based on RFCAB has a stronger obstacle avoidance ability. From Figure 5e, it is evident that the harvester *H#a*, located at the hollow region of the RFCAB‐based MWPT system, exhibits lower DC‐to‐DC transmission efficiency compared to the focused beam‐based MWPT system. This observation highlights the hollow characteristics of the RFCAB. To further validate the characteristics of the MWPT system based on ADCMs generating the RFCAB, a transmission system was implemented, as shown in Figure 5f. A vector network analyzer was used to measure the S_21_ parameter between the UCA and harvester, from which the DC‐to‐DC transmission efficiency, *η* = |S_21_|^2^, was calculated. Initially, the transmission efficiency measured at the RFCAB focal point by a single harvester (*H#r*) was 6.18%, and then another harvester (represented by *H#a*) was added. The corresponding results for the transmission efficiency are shown in Figure 5g,h. The results indicate that the inclusion of obstacles (represented by *H#a*) has minimal effect on the energy transmission efficiency at the focus of the RFCAB. Furthermore, the energy harvest efficiency of *H#a* within the hollow area is also quite low. These findings are in strong agreement with the simulation results, thus validating the characteristics of the RFCAB‐based MWPT system.

## 3 Conclusion

This study innovatively proposes an advanced MWPT system based on the RFCAB. To simplify the complexity of the beam generation system and ensure the efficient generation of RFCAB, high‐performance ADCMs based on the caustic theory has been designed and fabricated. For meeting the amplitude excitation dependent upon by ADCMs, a highly integrated UCA is designed for generating OAM beams as the optimal feed of the array. Meanwhile, the impact of UCA design and OAM mode on RFCAB generation is thoroughly analyzed. The simulation and experimental results demonstrate that the designed MWPT system can bypass obstacles in the environment and provide a safe area for high‐power microwave transmission. The characteristics has been further validated through comparison with other power transfer systems. The proposed MWPT system based on ADCMs generating RFCAB will advance the research and development of circular Airy beams in the radio frequency domain. It also provides a feasible solution to challenges such as obstacles in the environment faced by MWPT.

## Conflict of Interest

The authors declare no conflict of interest.

## Supporting information



Supporting Information

## Data Availability

The data that support the findings of this study are available from the corresponding author upon reasonable request.
