# Supplementary material for: Obstacle‐Immune Microwave Wireless Power Transfer System Based on Amplitude‐Dependent Caustic Metasurface
Source: Adv Sci (Weinh). 2025 Aug 4;12(39):e10070. doi: 10.1002/advs.202510070 (PMC12533293; doi:10.1002/advs.202510070)
Supplement: Supplementary file 1 — Supporting Information [file ADVS-12-e10070-s001.docx]

Supporting Information

Obstacle-Immune Microwave Wireless Power Transfer System Based on Amplitude-Dependent Caustic Metasurface

Song Zhang^1,+^, Hao Xue^1,+^, Zhe Zheng^1^, Xiangjin Ma^1^, Xin Wang^2,^, Jiaqi Han^1,+^, Haixia Liu^1,^*, and, Long Li^1,^*

^1^Key Laboratory of High-Speed Circuit Design and EMC of Ministry of Education, School of Electronic Engineering, Xidian University, Xi'an 710071, China

^2^School of Electronic Engineering, Xi’an University of Posts and Telecommunications, Xi’an 710100, China

^*^ Corresponding authors E-mail: [hxliu@xidian.edu.cn](mailto:hxliu@xidian.edu.cn) and [lilong@mail.xidian.edu.cn](mailto:lilong@mail.xidian.edu.cn)

This supporting information includes:

**Supplementary Notes 1-7**

**Figures S1-S16**

**Tables S1-S8**

**Supplementary Note 1. Caustic Phase of ADCMs for Generating RFCAB**

Initially, the Airy beam with arbitrary curvature, generated based on caustic theory and geometric optics, is analyzed. Following this, the RFCAB with arbitrary curvature, derived using the same theoretical framework, is discussed. The expression for the self-bending Airy beam is shown in Equation (S1), where *a* represents the curvature of the beam. The one-dimensional (1D) array that generates the beam is positioned along the *x_0_* axis, and the self-bending beam propagates in the *xoz*-plane, as illustrated in **Figure S1**, where the beam trajectory is represented by the red curve. According to caustic theory and geometric optics ^[1-2]^, the self-bending beam is an envelope formed by rays that are tangent to the trajectory emitted by the array unit, with these rays depicted by the black dashed line in **Figure S1**. The slope of these rays can be obtained by differentiating the expression for the curved trajectory, as shown in Equation (S2).


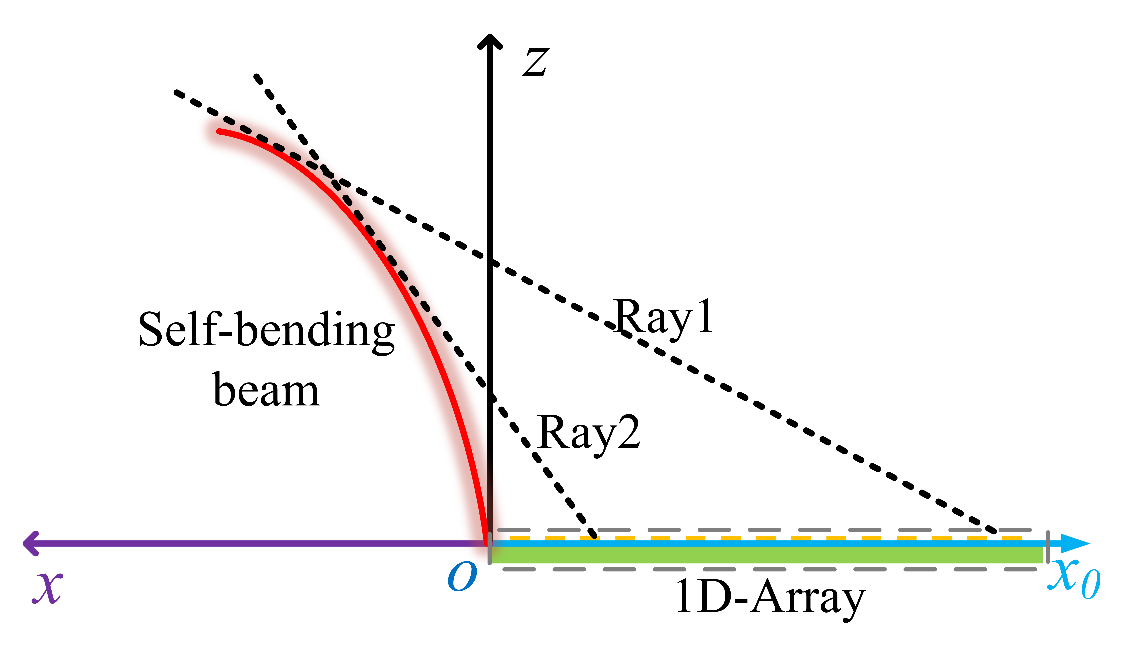


**Figure S1.** The schematic diagram for the generation of a self-bending Airy beam based on the caustic theory and geometric optics in the 1D case.

$x=az^{2}$ (S1)

$\frac{dz}{dx}=\frac{1}{2\sqrt{ax}}$ (S2)

Meanwhile, the slope of the isophase line at each unit of the array must be perpendicular to the emitted rays, as shown in Equation (S3) ^[3]^.

 (S3)

By integrating Equation (S3), the phase required for each unit of a 1D-array to generate a self-bending Airy beam can be determined:

 (S4)

where *k_0_* = 2π/λ (λ is the wavelength of the electromagnetic wave) is the wave number of the electromagnetic wave, and *x_0_* is the coordinate of the unit. Using Equation (S4), the phase distribution of the self-bending Airy beams with varying curvatures (corresponding to different values of *a*) can be obtained. The electric field distributions of Airy beams with various curvatures are shown in **Figure S2**.


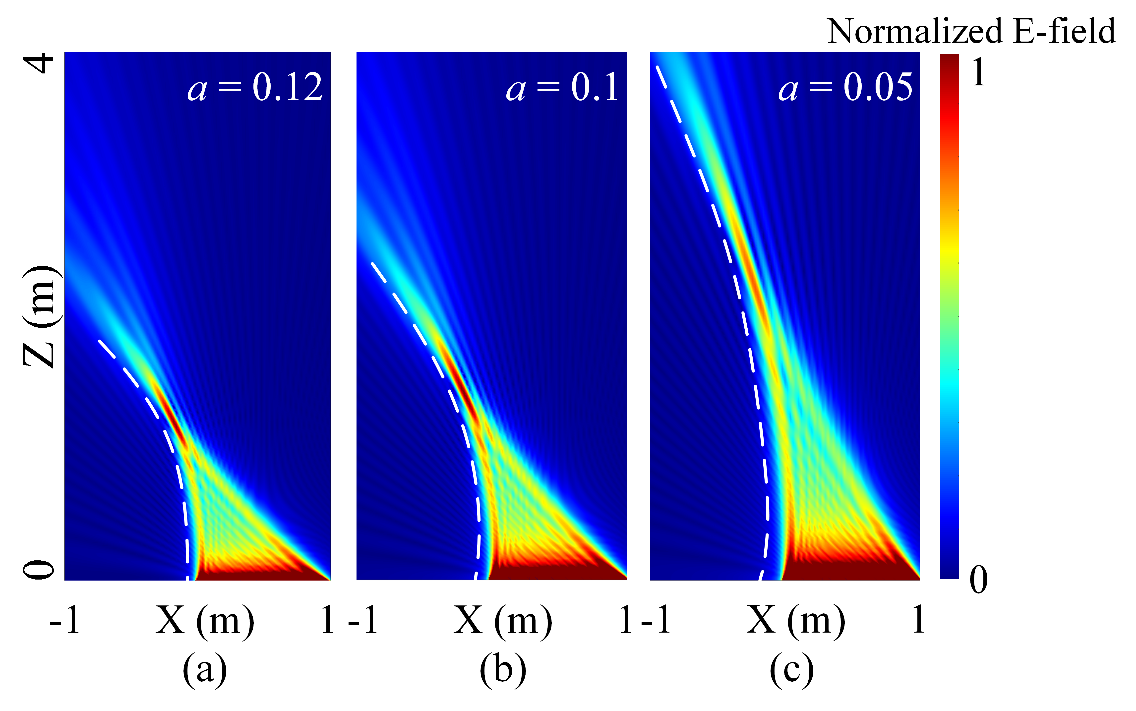


**Figure S2.** The normalized electric field (E-field) distributions of Airy beams with different values of *a*, where the white dashed line represents the trajectory of the beam.

The radio-frequency circular Airy beam (RFCAB) can be viewed as a radial superposition of multiple self-bending 1D Airy beams, resulting in a caustic surface that converges to an energy focus at the endpoint. Additionally, to ensure that the RFCAB, designed based on caustic theory and geometric optics, includes a hollow region (for safety and obstacle avoidance), the metasurface array must feature a dark disk region that generates extremely low beam energy, and the radius of the dark disk region is denoted as *r_0_*. Therefore, the phase required for each unit of the array to generate an RFCAB is a further expression of Equation (S4), given as follows:

 (S5)

where (*x_m_*,*y_m_*) represent the coordinates of the array unit. By substituting *r_0_* into Equation (S1), the distance *z_f_* between the focal point of the RFCAB and the metasurface array can be approximated, as given by Equation (S6). Furthermore, the size of the hollow region of the RFCAB can be characterized by Equation (S7) based on the volume fractions in cylindrical coordinates.

$z_{f}\approx\sqrt{r_{0}/a}$ (S6)

$V_{Hollow}=\int_{0}^{\sqrt{r_{0}/a}} \int_{0}^{2\pi} \int_{0}^{r_{0}} \pi r^{2}drd\theta dz$ (S7)

where *θ*∈[0,2π] of Equation (S7) represents the azimuth angle. From Equations (S6) and (S7), different values of *r_0_* and *a* will affect the focal length (*z_f_*) and the hollow region size (*V_Hollow_*) of the RFCAB.

**Supplementary Note 2. Design Principles of the UCA**

The uniform circular array (UCA) has been widely used to generate RF vortex electromagnetic beams with orbital angular momentum (OAM) ^[4-6]^. A vortex electromagnetic wave with a specified OAM mode order can be generated by loading equal-amplitude, equal-phase-difference feeds in a UCA with N units. The phase of each unit in the array can be determined as follows ^[6]^:

$P_{uu\_n}=l\psi_{n}$ (S8)

where $\psi_{n}=2\pi n/N$ is the azimuth angle of the *n*-th unit, and *l* is the mode order of the OAM beam. The diagram of a uniform circular array is shown in **Figure S3**.


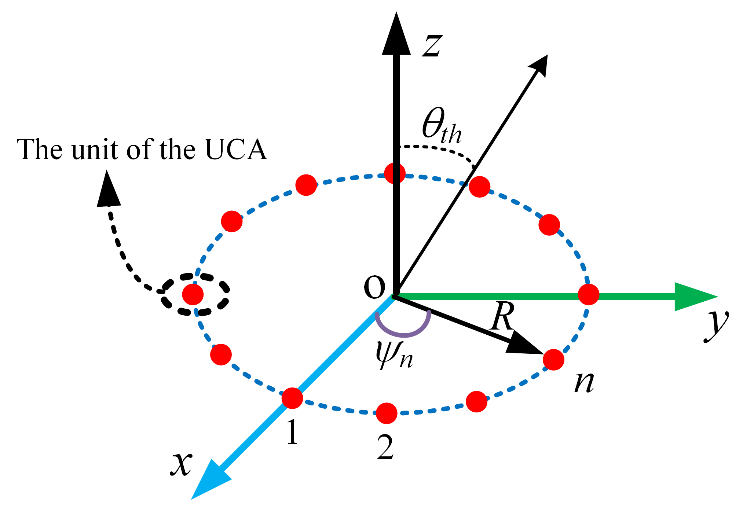


**Figure S3.** The diagram of a UCA, where *θ_th_* is the radiation direction of the beam and *R* represents the radius of the UCA.

By superimposing the radiation fields of each unit, the radiation amplitude distribution of the UCA array can be approximated ^[6]^:

$U\approx E_{0}^{2}J_{l}^{2}(k_{0}Rsin(\theta_{th}))$ (S9)

where *E_0_* is a constant independent of position, *J_l_* is the first kind Bessel function of order *l*, *R* is the radius of the circular array, and *θ_th_* is the pitch angle in the radiation direction. From Equation (S9), it can be seen that both the radius (*R*) of the array and the mode (*l*) of the OAM beam influence the distribution of the radiation intensity. **Figure S4**a illustrates the relationship between the radiation amplitude and the mode (*l*) when the array radius *R* = 2λ, where λ is the wavelength. **Figure S4**b shows the relationship between the radiation amplitude and the radius (*R*) when the mode is *l* = +1. The operating frequency of the array is 10 GHz.


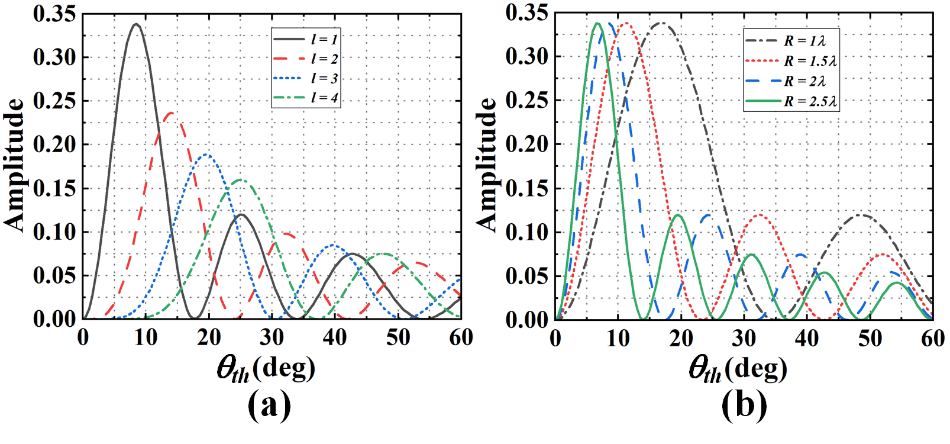


**Figure S4.** a) The radiation intensity of a UCA at different modes *l*. b) The radiation intensity of a UCA with different *R* values.

It can be seen from **Figure S4** that the beam divergence angle increases with the increase of the OAM mode order (*l*) when the UCA array radius (*R*) is constant. And, when the OAM mode (*l*) is constant, the beam divergence angle decreases with the increase of array radius (*R*). This satisfies the propagation characteristics of the OAM. Furthermore, along with the changes in the divergence angle caused by variations in the radius (*R*) or OAM mode order (*l*), the radiation amplitude distribution also undergoes changes. This behavior can be leveraged to tailor and manipulate the required amplitude distribution for generating RFCAB.

**Supplementary Note 3. Generation of RFCAB under different combinations of *R* and *l***

Here, an ADCMs driven by a UCA generating the OAM beams will be simulated and calculated in MATLAB for the generation of the RFCAB. And, the influence of the UCA radius (*R*) and the OAM beam mode order (*l*) on generated RFCAB will be discussed. The ADCMs array is designed to consist of 51×51 units, with a unit period of 10 mm. Moreover, the radius of the dark disk region of the array is set as *r_0_* = 75 mm, and the coefficient *a* of the RFCAB parabolic trajectory is 0.02. So, the caustic phase distribution required to generate the RFCAB can be obtained from Equation (S5), while the dependent excitation amplitude of the ADCMs corresponds to the amplitude distribution of the OAM beam generated by the UCA at a certain distance. The radiation field distribution of the ADCMs can be obtained by substituting the excitation amplitude and phase of the array into the spherical wave superposition method Equation (S10), as follows:^[7]^

 (S10)

where *Am_i_* and *φ_i_* are the excitation amplitude and phase of the *i*-th metasurface unit. *r_d_* represents the distance between the array unit and the field point.

Firstly, the impact of the UCA with varying *R* values, acting as the feed source for the array, in generating the RFCAB is investigated. It is assumed that the UCA is positioned at a distance of z = 300 mm from the ADCMs array and generates an OAM wave of *l* = 2 mode. The UCA is composed of 16 units that operate at 10 GHz, as same the metasurface. **Figure S5** illustrates the field distribution of the RFCAB on the vertical observation plane generated based on the proposed method, with the UCA at different *R* values acting as the driven source of the metasurface. Furthermore, **Figure S6** shows the effect of the ADCMs driven by OAM waves in different modes (*l*) emitted by the UCA on the generation of RFCAB. The distance between the metasurface and the UCA is set to z = 240 mm, and the radius of the UCA is *R* = 0.9 λ.


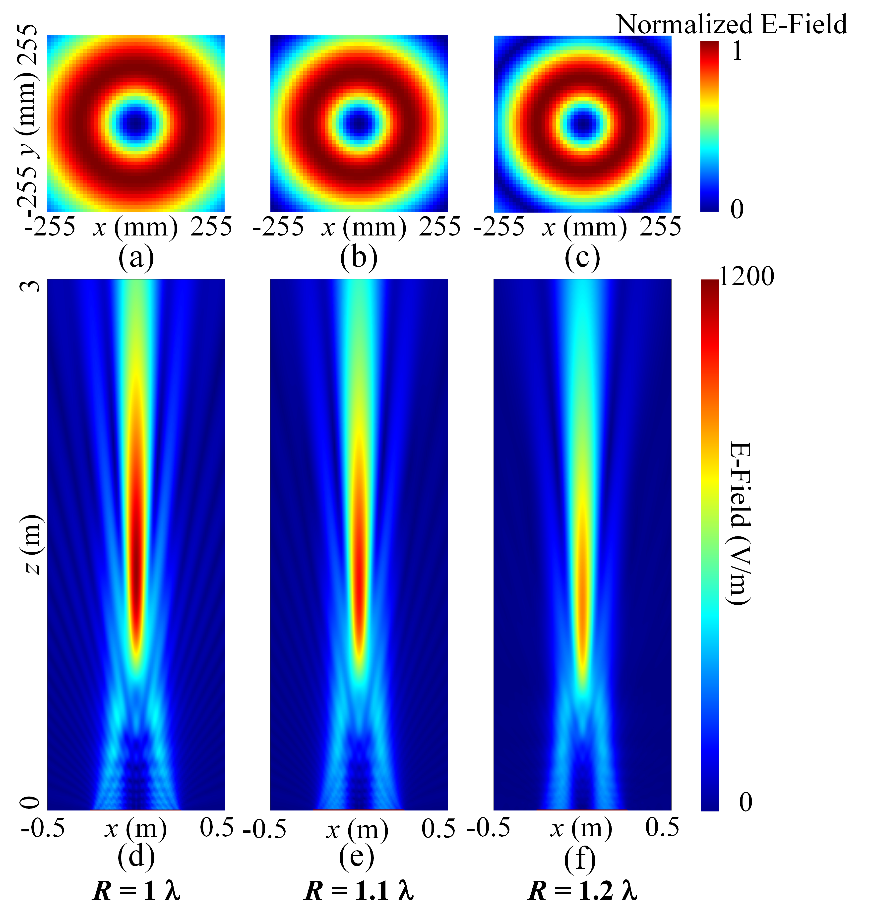


**Figure S5**. a-c) The electric field (E-field) distribution on the ADCMs of the OAM beams emitted by UCA with *R* = 1 λ, 1.1 λ, and 1.2 λ. d-f) The E-field distribution of the RFCAB on the vertical observation plane generated by the ADCMs excited by the OAM wave emitted by UCA with different *R* = 1 λ, 1.1 λ, and 1.2 λ. Where λ is the wavelength of the electromagnetic wave.


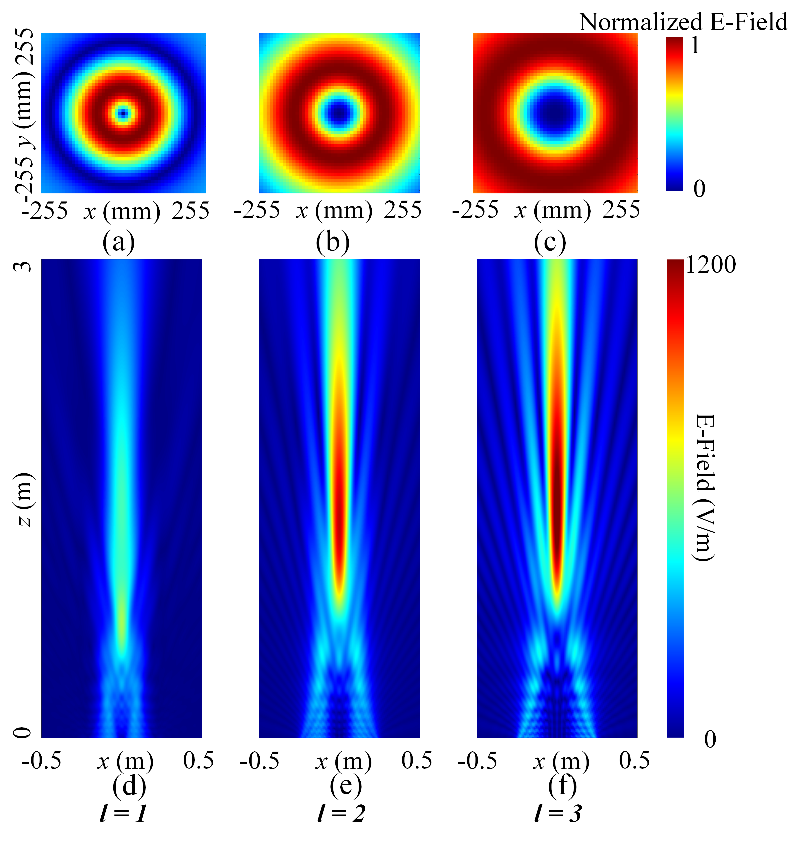


**Figure S6.** a-c) The normalized E-field distribution on the ADCMs of the OAM beams with *l* = 1, 2, and 3 emitted by the UCA. d-f) The E-field distribution of the RFCAB on the vertical observation plane generated by the metasurface excited by the OAM wave with different modes *l* = 1, 2, and 3 emitted by UCA.

**Supplementary Note 4. Simulation Setup for Generating focused and Bessel beams**

Similar to existing work on generating focused and Bessel beams based on the Gaussian beam ^[8-9]^, a horn antenna generating the approximating Gaussian beam is employed as the metasurface array excitation feed for the generation of the focused and Bessel beams based on the metasurface array. The scale of the array is consistent with that used for RFCAB generation. In this design, the distance between the horn antenna and the metasurface is set to *z_h_* = 0.8×D (D is the size of the metasurface) to enhance the energy utilization efficiency of the horn antenna. Assuming the horn antenna is oriented vertically to the array, the Gaussian amplitude distribution of the horn at the array can be calculated approximately according to Equation (S11) ^[10]^.

$AmH=\frac{cos^{q}\theta_{e}}{r_{f}}$ (S11)

where *q* is a constant, *θ_e_* is the pitch angle of the feed horn to the metasurface unit, and *r_f_* is the distance from the feed horn to the unit. Here, the value of *q* is selected as 6 to calculate the Gaussian excitation amplitude of the metasurface used to generate the focused beam and Bessel beam, as shown in **Figure S7**a.


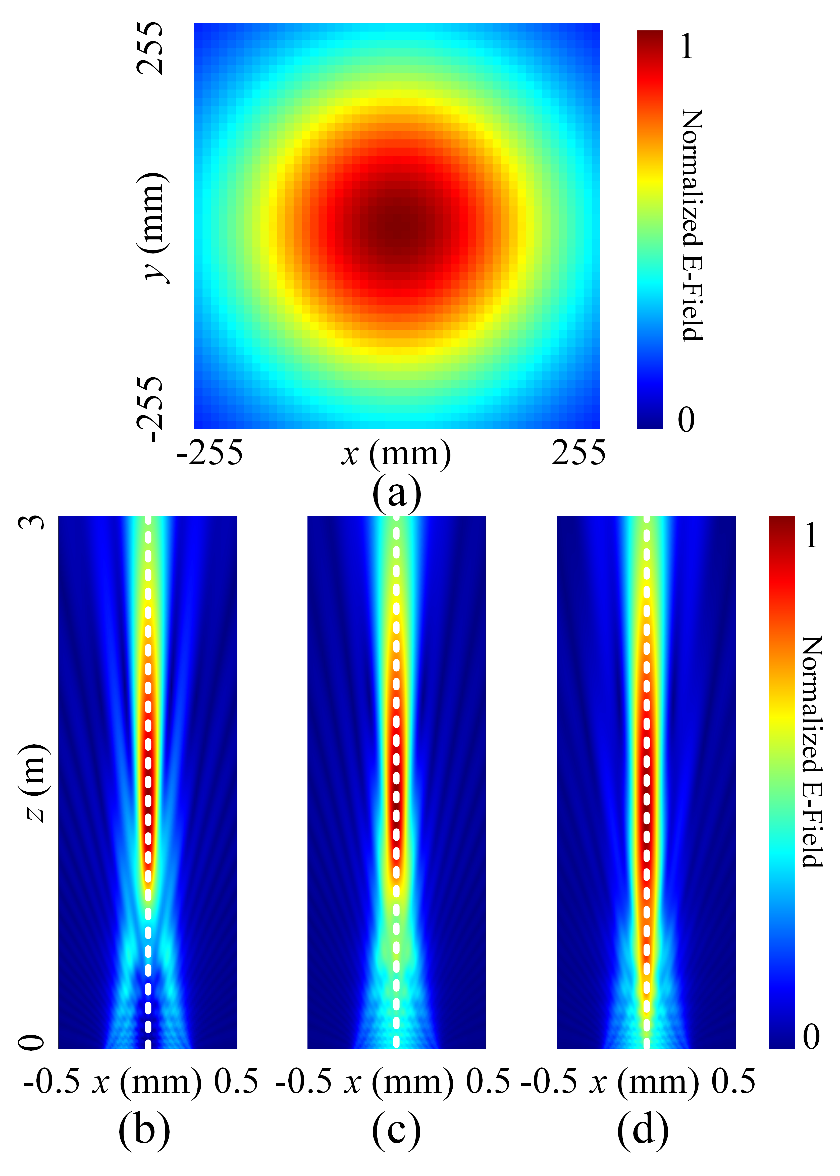


**Figure S7.** a) The excitation amplitude of the metasurface for the generation of the focused beam and Bessel beam. The normalized E-field distribution on the vertical plane of b) RFCAB, c) focused beam, and d) Bessel beam.

**Figure S7**b-d shows the normalized E-field distribution in the vertical plane for the RFCAB, focused beam, and Bessel beam, all generated using the same metasurface aperture. The focal point of the focused beam and the location of the strongest energy point of the Bessel beam coincide with that of the RFCAB. To demonstrate that RFCAB can provide a safe zone for high-power microwave transmission of MWPT, the field distribution along the path indicated by the white lines in **Figure S7**b-d is compared. **Figure S8** shows the variation in the normalized E-field along the white line as the variable z changes. As seen in the figure, before the field value along the white line reaches its maximum, there exists a hollow region where the RFCAB field value is lower than that of both the focused beam and the Bessel beam.


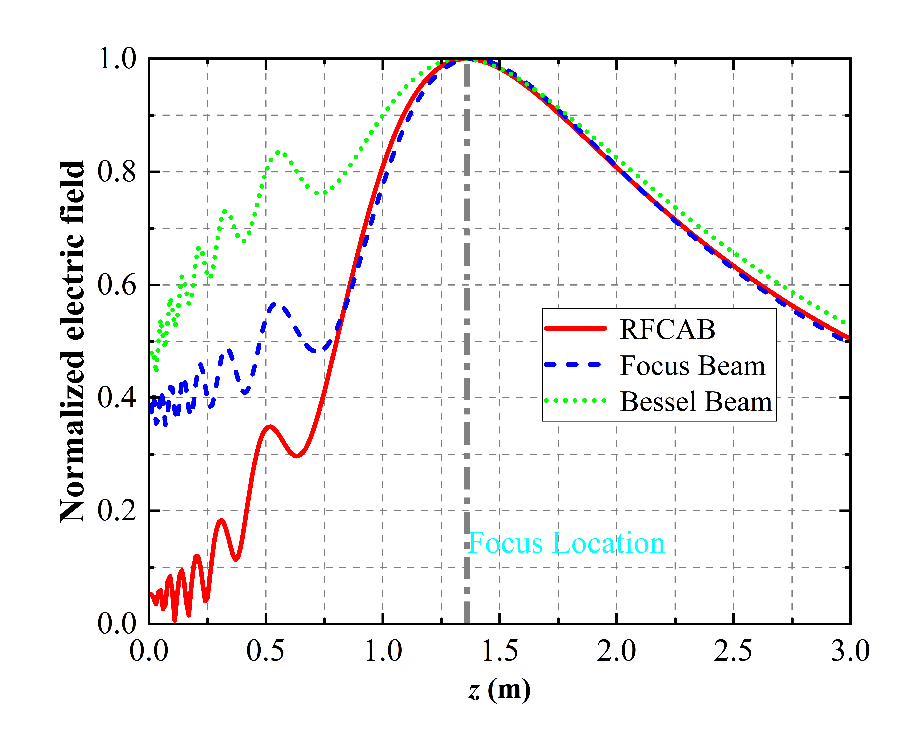


**Figure S8**. The change of the normalized field value with z on the white line in **Figure S7**b-d.

**Supplementary Note 5. Beams’ Power Transmission Analysis in FEKO**

By giving the excitation amplitude and phase of the metasurfaces that generate RFCAB, focused beam, and Bessel beam of **Figure S7**b-d to the current source in Altair FEKO, the impact of obstacles within the hollow region on beam transmission power can be efficiently assessed. The input power of the current source array is normalized to 1 W. Metal plates with the size of 100 mm × 100 mm and 140 mm × 140 mm are positioned at (0, 0, 150 mm), (0, 0, 250 mm), and (0, 0, 350 mm) above the arrays generating the RFCAB, focused beam, and Bessel beams, with the current source array located at z=0. Through simulation and calculation, the power distribution at the focal points of the three beams after the metal plate is added is shown in Tables S1 and S2.

**Table S1.** The power at (0,0,1.4 m) of the three beams without adding a metal plate obstacle, and after adding a metal plate with the size of 100 mm × 100 mm.

| Obstacle’s location | RFCAB | Focused Beam | Bessel Beam |
| --- | --- | --- | --- |
| No obstacle | 0.3489 W | 0.4132 W | 0.4015 W |
| (0,0,150 mm) | 0.3277 W | 0.3511 W | 0.3125 W |
| (0,0,250 mm) | 0.3176 W | 0.2793 W | 0.2391 W |
| (0,0,350 mm) | 0.2961W | 0.2694 W | 0.2282 W |

**Table S2**. The power at (0,0,1.4 m) of the three beams after adding the metal plate with a size of 140 mm × 140 mm.

| Obstacle’s location | RFCAB | Focused Beam | Bessel Beam |
| --- | --- | --- | --- |
| (0,0,150 mm) | 0.2959 W | 0.2998 W | 0.2569 W |
| (0,0,250 mm) | 0.2704 W | 0.1944 W | 0.1611 W |
| (0,0,350 mm) | 0.2377 W | 0.1800 W | 0.1506 W |

To further assess the obstacle’s influence on the power transmission of the beams, the metal plate is replaced with a wooden board, positioned as described above. Through simulation and calculation, the power distribution at the focal points of the three beams after the replacement of the wooden board is shown in **Tables S3 and S4**.

**Table S3.** The power at (0,0,1.4 m) of the three beams after adding a wooden board obstacle with a size of 100 mm × 100 mm.

| Obstacle’s location | RFCAB | Focused Beam | Bessel Beam |
| --- | --- | --- | --- |
| (0,0,150 mm) | 0.3348 W | 0.3847 W | 0.3137 W |
| (0,0,250 mm) | 0.3246 W | 0.3755 W | 0.3011 W |
| (0,0,350 mm) | 0.3076 W | 0.3617 W | 0.2927 W |

**Table S4.** The power at (0,0,1.4 m) of the three beams after adding a wooden board obstacle with a size of 140 mm × 140 mm.

| Obstacle’s location | RFCAB | Focused Beam | Bessel Beam |
| --- | --- | --- | --- |
| (0,0,150 mm) | 0.3234 W | 0.3677 W | 0.2890 W |
| (0,0,250 mm) | 0.3060 W | 0.3514 W | 0.2716 W |
| (0,0,350 mm) | 0.2905 W | 0.3377 W | 0.2658 W |

To better demonstrate the obstacle avoidance performance of RFCAB, the improvement index (EI) is defined to quantify the improvement rate of the obstacle avoidance capability of the MWPT system based on RFCAB when facing obstacles in the environment compared with the MWPT systems of focused beams and Bessel beams. The calculation method of EI is shown in Equation (S12).

$EI=(\frac{P_{Robs}}{P_{R0}}\div\frac{P_{FBobs}}{P_{FB0}}-1)\times100\%$ (S12)

Where *P_Robs_* represents the power at the RFCAB focus in the presence of an obstacle, while *P_R0_* denotes the power at the RFCAB focus in the absence of obstacles. Similarly, *P_FBobs_* indicates the power at the focus of the focused beam or Bessel beam with obstacles, and *P_FB0_* represents the power at the focus of the focused beam or Bessel beam without obstacles. By substituting the values from **Tables S1-S4** into Equation (S12), the EI of RFCAB relative to the focused beam and Bessel beam at various obstacle positions and sizes can be obtained, as shown in **Tables S5-S8**.

**Table S5.** The EI of the RFCAB relative to the focused beam and Bessel beam after adding a metal plate obstacle with a size of 100 mm × 100 mm at various locations.

| Obstacle’s location | EI of RFCAB-Focused Beam | EI of RFCAB-Bessel Beam |
| --- | --- | --- |
| (0,0,150 mm) | 10.5% | 20.67% |
| (0,0,250 mm) | 34.68% | 52.86% |
| (0,0,350 mm) | 30.17% | 49.31% |

**Table S6.** The EI of the RFCAB relative to the focused beam and Bessel beam after adding a metal plate obstacle with a size of 140 mm × 140 mm at various locations.

| Obstacle’s location | EI of RFCAB-Focused Beam | EI of RFCAB-Bessel Beam |
| --- | --- | --- |
| (0,0,150 mm) | 16.88% | 32.5% |
| (0,0,250 mm) | 64.72% | 93.17% |
| (0,0,350 mm) | 56.4% | 81.6% |

**Table S7.** The EI of the RFCAB relative to the focused beam and Bessel beam after adding a wooden board obstacle with a size of 100 mm × 100 mm at various locations.

| Obstacle’s location | EI of RFCAB-Focused Beam | EI of RFCAB-Bessel Beam |
| --- | --- | --- |
| (0,0,150 mm) | 2.86% | 17.83% |
| (0,0,250 mm) | 2.16% | 18.05% |
| (0,0,350 mm) | 0.62% | 15.26% |

**Table S8.** The EI of the RFCAB relative to the focused beam and Bessel beam after adding a wooden board obstacle with a size of 140 mm × 140 mm at various locations.

| Obstacle’s location | EI of RFCAB-Focused Beam | EI of RFCAB-Bessel Beam |
| --- | --- | --- |
| (0,0,150 mm) | 3.7% | 20.71% |
| (0,0,250 mm) | 2.66% | 20.05% |
| (0,0,350 mm) | 1.53% | 17.06% |

**Supplementary Note 6. The unit of UCA and Array Design**

The unit of the UCA is shown in **Figure S9**, and the size parameters of the unit are shown in **Table S9**. The top metal patch of the unit has dimensions *L*=8 mm and *w* = 8.78 mm along the *x*- and *y*-axes, respectively, while the substrate height is *hsub*=1 mm. The feed point on the patch is located *F_y_*=1.3 mm from the patch center along the *y*-direction, ensuring the generation of a *y*-polarization radiation pattern, as shown in **Figure S10**. The radius of the metallization hole for the feed is *F_r_*=0.45 mm.


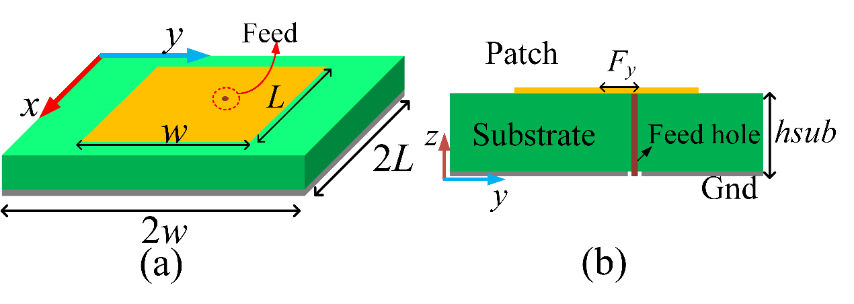


**Figure S9.** The unit of the UCA. a) The overview of the unit. b) The side view of the unit.


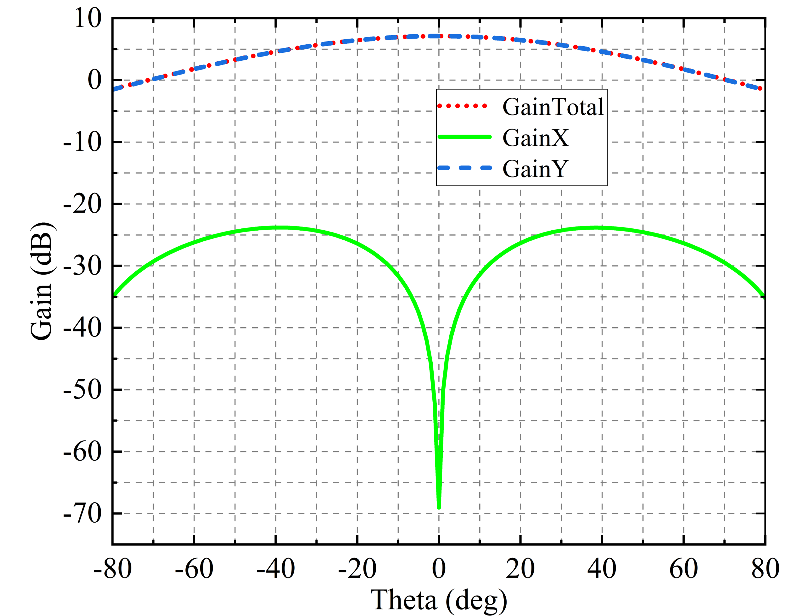


**Figure S10.** The radiation pattern of the designed UCA unit.

**Table S9.** The design parameters of the UCA unit.

| Parameter | Value |
| --- | --- |
| *w* | 8.78 mm |
| *L* | 8 mm |
| *hsub* | 1 mm |
| *Fy* | 1.3 mm |
| *F_r_* | 0.45 mm |


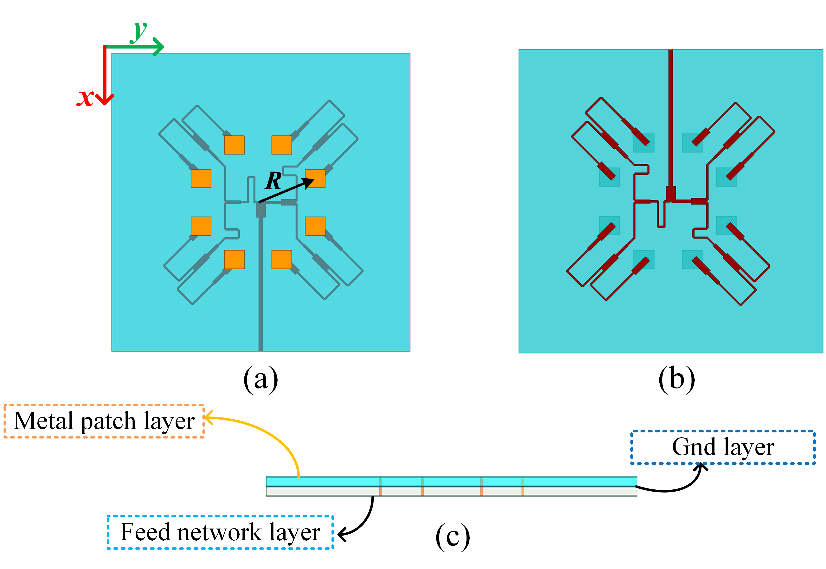


**Figure S11.** a) The top view, b) the bottom view, c) the side view of the UCA.

In order to simplify the design of the UCA array that generates *l*=-2 mode OAM waves, 8 units are used here to discretize the required phase, with the phase of each unit calculated according to Equation (S8). The designed UCA array is shown in **Figure S11**.

To validate the correctness of the design, the UCA prototype was fabricated and measured in a microwave anechoic chamber, as shown in **Figure S12**.


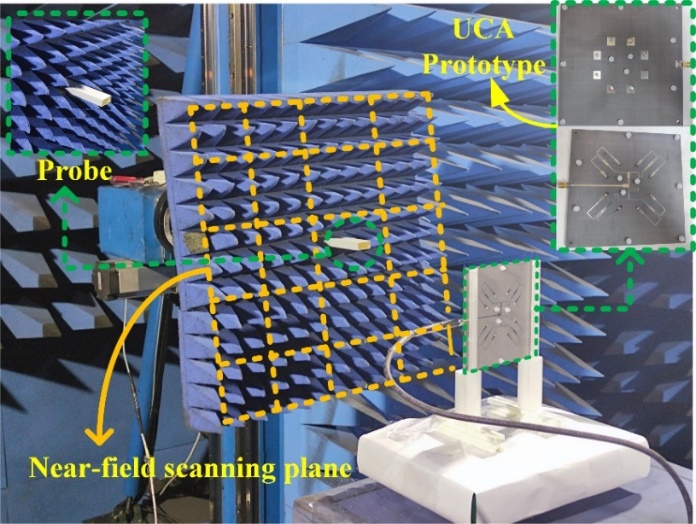


**Figure S12.** The microwave anechoic chamber measurement scenario and the prototype of the designed UCA.

**Supplementary Note 7. The Array Design and Simulation of ADCMs**

Since the UCA is 240 mm away from the ADCMs for providing the necessary amplitude excitation, the path delay phase caused by the feed position needs to be compensated by the units according to Equation (S13). The phase distribution is shown in **Figure 4**d (***ϕ_2_***).

$PD_{i,j}=k_{0}\sqrt{(x_{ij}-x_{F})^{2}+(y_{ij}-y_{F})^{2}+(z_{ij}-z_{F})^{2}}$ (S13)

where (*x_ij_*,*y_ij_*,*z_ij_*) are the spatial coordinates of the unit, (*x_F_*,*y_F_*,*z_F_*) represent the spatial coordinates of the feed. The phase distributions required for designing the ADCMs are shown in **Figure 4**. Based on the relationship between phase and unit parameters, the ADCMs can be constructed as shown in **Figure S13.**


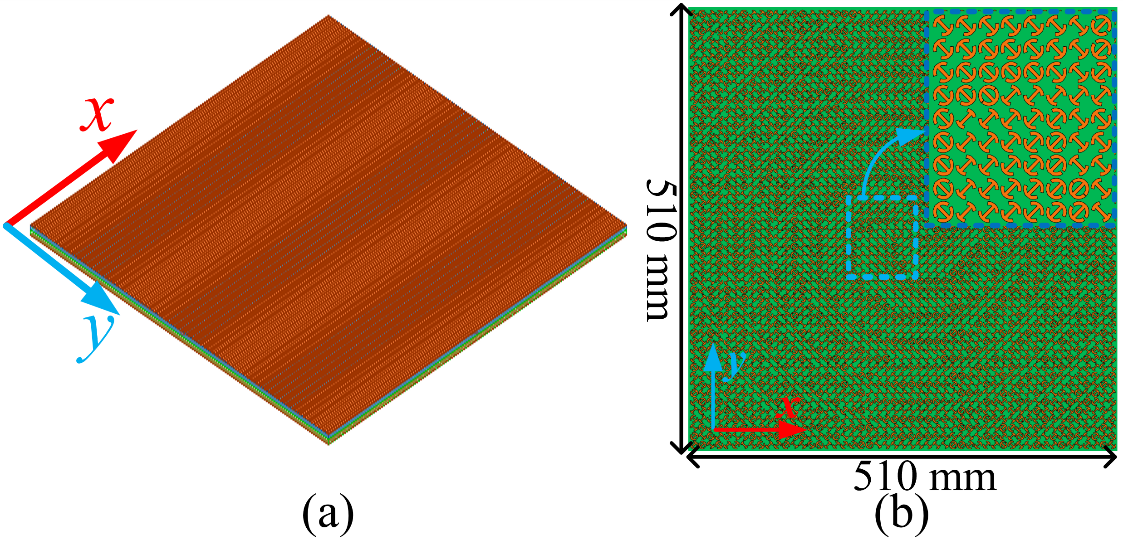


**Figure S13.** a) The constructed ADCMs. b) The middle metal patch layer of the ADCMs.

Based on the designed ADCMs and UCA models, an efficient RFCAB generation system is composed, and the normalized electric field distribution of RFCAB on the vertical plane can be obtained through simulation in ANSYS HFSS, as shown in **Figure S14**.


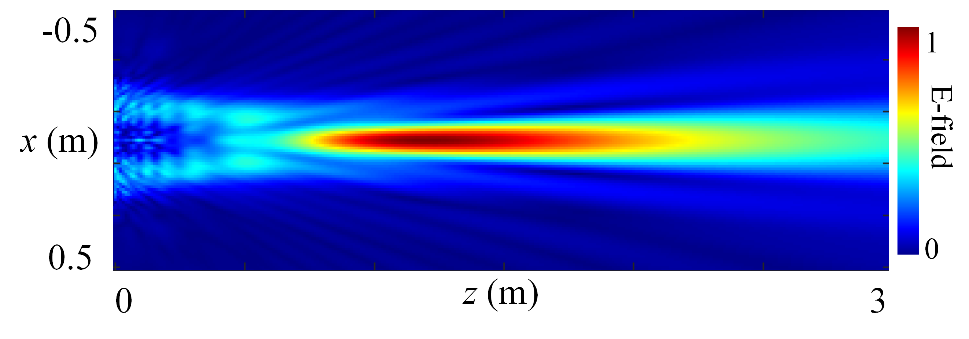


**Figure S14.** The simulated normalized electric field distribution of RFCAB on the vertical plane.

For verifying the correctness of the design, the prototype of the RFCAB generation system was fabricated and measured in a microwave anechoic chamber, as shown in **Figure S15**.


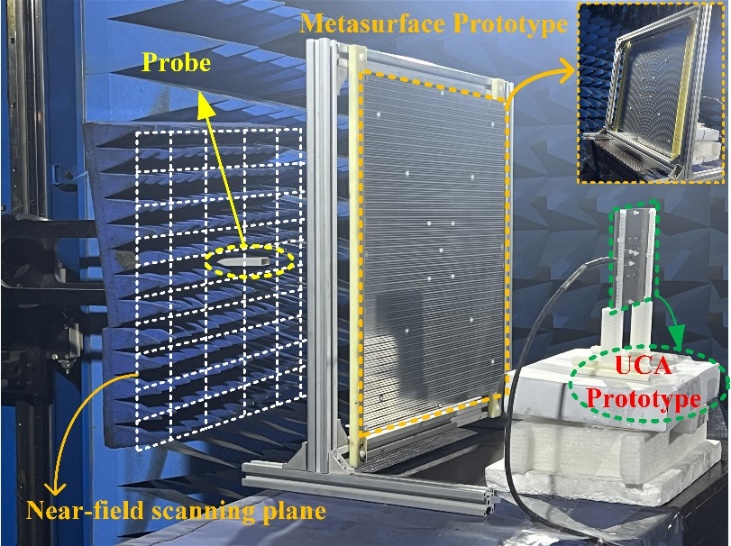


**Figure S15.** The microwave anechoic chamber measurement scenario of the prototype of the RFCAB generation system.

Moreover, an advanced obstacle-immune MWPT system based on ADCMs generating RFCAB is constructed by adding an energy harvester composed of 2×2 microstrip antennas at the focal point (*z_f_* = 1400 mm). **Figure S16**a is the designed microstrip antenna array harvester, and its size is 50 mm×50 mm. From **Figure S16**b, the reflection coefficient of the harvester shows a good match. The schematic diagram of the constructed MWPT system is shown in **Figure S16**c.


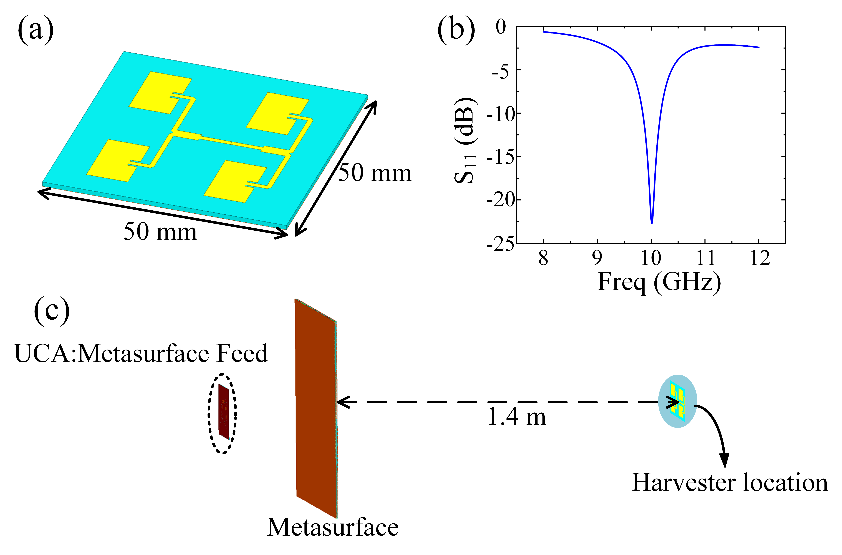


**Figure S16.** a) The model diagram of the harvester based on the microstrip antenna array. b) The S11 curve of the harvester. c) The diagram of the constructed MWPT system based on the ADCMs generating the RFCAB.

**References**

1. Y. Qian, L. Dong, H. Mao, *IEEE Photonics J.* **2017**, 9, 6101411.
2. I. D. Chremmos, G. Fikioris, N. K. Efremidis, *IEEE Trans. Antennas Propag.* **2013**, 61, 5048.
3. D. M. Cottrell, J. A. Davis, T. M. Hazard, *Opt. Lett.* **2009**, 34, 2634.
4. B. Thidé, H. Then, J. Sjöholm, K. Palmer, J. Bergman, T. D. Carozzi, Ya. N. Istomin, N. H. Ibragimov, R. Khamitova, *Phys. Rev. Lett.* **2007**, 99, 087701.
5. S. M. Mohammadi, L. K. S. Daldorff, J. E. S. Bergman, R. L. Karlsson, B. Thidé, K. Forozesh, T. D. Carozzi, B. Isham, *IEEE Trans. Antennas Propag.* **2010**, 58, 565.
6. T. Yuan, Y. Cheng, H. Wang, Y. Qin, *IEEE Trans. Antennas Propag.* **2017**, 65, 688.
7. Y. Huang, X. Li, Z. Akram, H. Zhu, Z. Qi, *IEEE Antennas Wireless Propag. Lett.* **2021**, 20, 1093.
8. H. Xue, X. Wu, X. Cui, M. Chang, H. Liu, L. Li, T. J. Cui, *IEEE Trans. Microw. Theory Techn.* **2022**, 70, 4449.
9. X. Wu, F. Hou, Y. Li, S. Zhao, S. Zhang, H. Xue, M. Chang, J. Han, H. Liu, L. Li, *IEEE Trans. Microw. Theory Techn.* **2023**, 71, 3479.
10. P. Nayeri, F. Yang, A. Z. Elsherbeni, *Reflectarray Antennas: Theory, Designs, and Applications*, Wiley, Hoboken, NJ, USA **2018**.
